# The Mitochondrial NAD Transporter SLC25A51 in Adipocytes Regulates Adipose Tissue Mitochondrial Function and Systemic Metabolism During Aging

**DOI:** 10.1111/acel.70509

**Published:** 2026-04-21

**Authors:** Daiki Kojima, Keisuke Yaku, Shotaro Kosugi, Ryunosuke Mitsuno, Kenji Kaneko, Yoshinaga Kawano, Akihito Hishikawa, Seiya Mizuno, Manami Katoh, Akiko Satoh, Shinya Toyokuni, Tatsuhiko Azegami, Kenichiro Kinouchi, Shintaro Yamaguchi, Hiroshi Itoh, Takeshi Kanda, Toshimasa Yamauchi, Takashi Nakagawa, Kaori Hayashi, Jun Yoshino

**Affiliations:** ^1^ Division of Endocrinology, Metabolism and Nephrology, Department of Internal Medicine Keio University School of Medicine Tokyo Japan; ^2^ Department of Molecular and Medical Pharmacology, Faculty of Medicine University of Toyama Toyama Japan; ^3^ Laboratory Animal Resource Center in Transborder Medical Research Center, Institute of Medicine University of Tsukuba Tsukuba Japan; ^4^ Departments of Cardiovascular Medicine The University of Tokyo Tokyo Japan; ^5^ Department of Frontier Cardiovascular Science The University of Tokyo Tokyo Japan; ^6^ Department of Integrative Physiology, Division of Brain Science, Institute of Development, Aging and Cancer (IDAC) Tohoku University Sendai Japan; ^7^ Department of Integrative Physiology Geroscience Research Center, National Center of Geriatrics and Gerontology Obu Japan; ^8^ Department of Pathology and Biological Responses Nagoya University Graduate School of Medicine Nagoya Japan; ^9^ Center for Preventive Medicine Keio University Hospital Tokyo Japan; ^10^ Division of Nephrology, Department of Internal Medicine Shimane University Izumo Shimane Japan; ^11^ The Center for Integrated Kidney Research and Advance (IKRA), Faculty of Medicine Shimane University Izumo Shimane Japan; ^12^ Department of Diabetes and Metabolic Diseases, Graduate School of Medicine The University of Tokyo Tokyo Japan

**Keywords:** adipocyte, aging, insulin resistance, NAD, obesity

## Abstract

Nicotinamide adenine dinucleotide (NAD) is a classical coenzyme regulating cellular energy metabolism. Emerging evidence demonstrates the causal relationship between defective NAD metabolism and various age‐associated diseases. The major purpose of the present study was to investigate the role of adipocyte mitochondrial NAD biology in age‐associated metabolic diseases. To this end, we focused on solute carrier family 25 member 51 (SLC25A51), a recently identified mitochondrial NAD transporter. We found that aging was associated with decreased adipose tissue SLC25A51 expression in both humans and mice. We next generated and analyzed novel knockout and overexpression models, which we have named adipocyte‐specific *Slc25a51* knockout (ASKO) and *Slc25a51* overexpressing (ASLO) mice. ASKO mice had a marked decrease in adipose tissue mitochondrial NAD levels and exhibited age‐associated systemic metabolic complications, such as obesity, glucose intolerance, insulin resistance, hyperinsulinemia, metabolic inflexibility, dyslipidemia, and hepatosteatosis. Mechanistically, loss of *Slc25a51* reduced mitochondrial respiratory function, fatty acid oxidation capacity, and adiponectin production in adipose tissue, likely contributing to the development of systemic metabolic complications. Conversely, ASLO mice were protected from obesity and insulin resistance caused by aging. In conclusion, our results provide novel mechanistic and therapeutic insights into understanding the critical role of adipocyte mitochondrial NAD transporter SLC25A51 in the pathophysiology of age‐associated metabolic diseases, particularly obesity and insulin resistance.

## Graphical abstract

We revealed the pathophysiological significance and therapeutic potential of adipocyte mitochondrial NAD transporter, namely solute carrier family 25 member 51 (SLC25A51), in age‐associated metabolic diseases, particularly adipose tissue mitochondrial function, obesity, and insulin resistance, by analyzing adipocyte‐specific *Slc25a51* knockout (ASKO) and *Slc25a51* overexpressing (ASLO) mice.

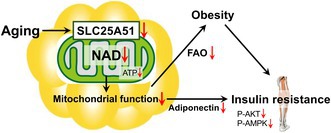

## 1 Introduction

Aging is associated with an increased risk of systemic metabolic diseases such as obesity, type 2 diabetes, insulin resistance, β‐cell dysfunction, and dyslipidemia (Palmer and Jensen 2022). The complex mechanisms responsible for age‐associated metabolic diseases remain unknown but could involve dysfunction of adipose tissue, a crucial metabolic and endocrine organ regulating whole‐body energy metabolism (Scherer 2016). Previous studies have revealed the close relationship between aging and adipose tissue mitochondrial dysfunction manifested by defects in mitochondrial fatty acid oxidation (FAO) and oxidative phosphorylation (Scherer 2016; Soro‐Arnaiz et al. 2016). In addition, mitochondrial dysfunction is causally involved in impairing key adipose tissue metabolic pathways, such as lipid synthesis, lipolysis, and adipokine production, thereby contributing to the development of systemic metabolic diseases (Ghaben and Scherer 2019; Kusminski and Scherer 2012).

Nicotinamide adenine dinucleotide (NAD) plays an essential role in regulating mitochondrial redox signals across species (Chini et al. 2023, 2016; Yoshino et al. 2018). Emerging evidence has revealed that NAD declines with aging in various tissues and defective NAD metabolism is involved in the pathophysiology of age‐associated cardiometabolic diseases such as type 2 diabetes, dyslipidemia, chronic kidney disease, and heart failure (Chini et al. 2016, 2023; Katsyuba et al. 2020; McReynolds et al. 2020; Yoshino et al. 2018, 2011). We and others have demonstrated that impaired NAD metabolism in white adipose tissue (WAT) causes systemic metabolic derangements including glucose intolerance, insulin resistance, dyslipidemia, hypoadiponectinemia, metabolic inflexibility, and mitochondrial dysfunction (Franczyk et al. 2021; Nielsen et al. 2018; Qi et al. 2024; Stromsdorfer et al. 2016; Yamaguchi et al. 2019). In addition, administration of nicotinamide mononucleotide (NMN), a key NAD intermediate, prevents adipose tissue inflammation, obesity, and insulin resistance caused by aging (Mills et al. 2016; Yoshino et al. 2018). Taken together, these findings shed light on the importance of adipose tissue NAD biology in age‐associated metabolic diseases. However, the role of adipose tissue mitochondrial NAD biology remains unexplored.

The major purpose of the present study was to investigate the role and therapeutic potential of adipose tissue mitochondrial NAD biology in age‐associated systemic metabolic diseases. To this end, we focused on solute carrier family 25 member 51 (SLC25A51), a recently identified mitochondrial NAD transporter (Girardi et al. 2020; Kory et al. 2020; Luongo et al. 2020). SLC25A51 selectively imports NAD into the mitochondrial matrix from the cytosol and critically regulates oxidative tricarboxylic acid (TCA) cycle activity and ATP production (Girardi et al. 2020; Kory et al. 2020; Luongo et al. 2020). However, the functional role of SLC25A51 in regulation of adipose tissue and whole‐body metabolic functions in vivo remains unclear. In the present study, we first evaluated the relationship among adipose tissue *SLC25A51* expression, aging, and insulin sensitivity in humans. To determine the role of adipose tissue mitochondrial NAD biology in the pathophysiology of age‐associated metabolic complications, we next generated and characterized novel knockout and overexpression models, which we have named adipocyte‐specific *Slc25a51* knockout (ASKO) and *Slc25a51* overexpressing (ASLO) mice respectively.

## 2 Methods

### 2.1 Animal Experimentation

C57BL/6J (B6) mice were purchased from the Jackson Laboratory Japan (Kanagawa, Japan). Old B6 mice were obtained from the NCGG Aging Farm (Aichi, Japan). Mice were housed under controlled light (12 h light/12 h dark) and had *ad libitum* access to standard diet and water. Blood was collected after a 6‐h fast for the measurements of insulin and adiponectin. At the endpoint, mice were anesthetized and tissue samples were harvested at fed conditions, frozen in liquid nitrogen, and stored at −80°C until analyses. All animal studies were approved by the Institutional Animal Care and Use Committees at Keio University and Shimane University.

### 2.2 Generation of Adipocyte‐Specific Slc25a51 Knockout (ASKO) and Overexpressing (ASLO) Mice

To generate floxed *Slc25a51* mice, two mouse genomic sequences (5′‐ATGTGATATTAACAGGCCAT‐3′) and (5′‐CAGTGCTCTGTCTGCAAGTT‐3′) in the intron 2 and 3′‐UTR of *Slc25a51* were selected as the sgRNA targets. The flox donor plasmid (pflox‐*Slc25a51*) contained the genomic region spanning 1802 bp upstream to 1028 bp downstream of the gene ORF. Two loxP sites were inserted at positions 576 bp upstream and 112 bp downstream of the ORF in this donor vector. The CRISPR‐Cas9 ribonucleoprotein complex together with donor DNA was microinjected into C57BL/6J zygotes (Jackson Laboratory Japan, Kanagawa, Japan). Adipocyte‐specific *Slc25a51* knockout (ASKO) mice were generated by crossing the floxed *Slc25a51* mice with adiponectin (*Adipoq*)‐Cre transgenic mice.

An *Adipoq* promoter was generated by joining two genomic regions on chromosome 16 (chr16:23,141,636–23,146,841 and chr16:23,155,017–23,155,257; GRCm38/mm10) (Wang et al. 2010). To generate adipocyte‐specific *Slc25a51* overexpressing (ASLO) mice, a Kozak sequence, *Slc25a51* cDNA, and the SV40 polyadenylation signal (SV40 pA) were inserted immediately downstream of this promoter to construct the transgene. The transgene was microinjected into male pronuclei of C57BL/6J zygotes. Surviving pronuclear‐stage embryos were subsequently transferred into the oviducts of pseudopregnant ICR females, yielding founder mice.

### 2.3 Adipocyte Isolation

Adipose tissue samples were incubated in Krebs Ringer buffer containing 1 mg/mL of collagenase type I (#LS004196; Worthington Biochemical, Lakewood, NJ) at 37°C for 45 min. After collagenase digestion, adipocytes were separated by centrifugation.

### 2.4 Glucose and Energy Metabolism

Mice were fasted for 6 h and 50% dextrose solution (2 g/kg‐body weight) was injected for intraperitoneal glucose tolerance tests (IPGTTs) as we previously described (Porter et al. 2018; Qi et al. 2024; Stromsdorfer et al. 2016; Yamaguchi et al. 2019, 2023). For insulin tolerance tests (ITTs), mice were injected with human insulin (0.75 U/kg‐body weight) after approximately 4 h of fasting. We measured metabolic parameters including insulin, adiponectin, free fatty acids (FFA), and triglyceride (TG) using the commercially available kits (Table S1). Oxygen consumption (VO_2_), CO_2_ production (VCO_2_), energy expenditure (EE), and respiratory quotient (RQ = VCO_2_/VO_2_) were determined using a biogas analytical mass spectrometer (ARCO‐2000; Arco System, Chiba, Japan). EE values were adjusted for body mass by Analysis of Covariance (ANCOVA; Banks et al. 2025).

### 2.5 RNA Isolation and Real‐Time PCR

Total RNA was isolated by using RNeasy mini kit (#74104; Qiagen, Chatsworth, CA) or TRIzol Reagent (#15596018; Invitrogen, Carlsbad, CA). The relative gene expression was determined by normalizing the Ct value to the housekeeping gene, ribosomal protein (36b4). Primer details are listed in Table S2. The composite expression levels of genes encoding proteins involved in FAO were calculated after expression of each gene to a Z‐distribution as we previously described (Mitsuno et al. 2025).

### 2.6 Adipose Tissue Respiration and Fatty Acid Oxidation

Oxygen consumption rates (OCR) were determined ex vivo with the Seahorse XFe24 Analyzer (Agilent Technologies, Santa Clara, CA). Approximately 4 mg of adipose tissue was placed into the XF24 Islet Capture Microplate (101122‐100, Agilent Technologies). Adipose tissue was treated with oligomycin (30 μM), FCCP (2 μM), and rotenone/antimycin A (5 μM). To evaluate the rate of FAO, we measured OCR in adipose tissue cultured in the presence or absence of palmitate (200 μM).

### 2.7 Metabolites Measurements

Mitochondria were isolated from fresh adipose tissues using the commercially available kit (#89801; Thermo Fisher Scientific, Rockford, IL). Samples were extracted by methanol‐chloroform. NAD and pentose phosphate pathway metabolites were analyzed using an Agilent 6460 Triple Quadrupole mass spectrometer coupled to an Agilent 1290 HPLC system (Agilent Technologies) with an Atlantis T3 column (Waters Corporation, Milford, MA). TCA metabolites were analyzed in selected ion monitoring mode using an Agilent 5977 MSD Single Quadrupole mass spectrometer coupled to an Agilent 7890 gas chromatograph (Agilent Technologies) as we previously described (Gulshan et al. 2018; Yaku et al. 2018; Yamaguchi et al. 2023). Data were analyzed using MassHunter Quantitative Analysis software (Agilent). ATP concentrations were determined by using the ATP measuring kit (#TA100; TOYO INK, Tokyo, Japan).

### 2.8 Western Blot Analysis

Proteins were extracted, loaded onto polyacrylamide gels, separated by SDS‐PAGE, and transferred to PVDF membranes as described previously (Porter et al. 2018; Stromsdorfer et al. 2016; Yamaguchi et al. 2019). The details of antibodies were provided in Table S3. Blot densitometry was quantitated using ImageJ software (NIH ImageJ 1.54; http://imagej.nih.gov/ij).

### 2.9 Insulin Signaling

Mice were fasted for 4 h and then intraperitoneally injected with a bolus of human insulin (1.0 U/kg body weight). After 10 min, tissues were harvested for western blot analysis.

### 2.10 Histological Assessment

Adipose tissue was fixed in paraformaldehyde and embedded in paraffin. Sections were stained with hematoxylin and eosin. We performed a quantitative analysis of lipid size as described previously (Mitsuno et al. 2025).

### 2.11 Mitochondrial DNA Content

DNA was extracted by using QIAamp DNA Mini Kit (#51034, Qiagen). Mitochondrial DNA content was evaluated by quantitating expression of mitochondria‐encoded gene (*16S rRNA*) and nuclear‐encoded gene (hexokinase 2, *Hk2*) as described previously (Porter et al. 2018; Yamaguchi et al. 2019).

### 2.12 SLC25A51 Expression in Human Adipose Tissue

We evaluated *SLC25A51* gene expression in human adipose tissue samples reported in publicly available microarray datasets obtained from the Gene Expression Omnibus (GEO) database (GSE27949; https://www.ncbi.nlm.nih.gov/geo/; Keller et al. 2011).

### 2.13 Statistical Analysis

Differences between two groups were assessed using Student's *t*‐test. Pearson's correlation analysis was used to examine correlations between outcomes of interest. Data are presented as means ± SEM. A *p* value < 0.05 was considered statistically significant.

## 3 Results

### 3.1 Aging Was Associated With Decreased Adipose Tissue SLC25A51 Expression in Humans and Mice

**FIGURE 1:**
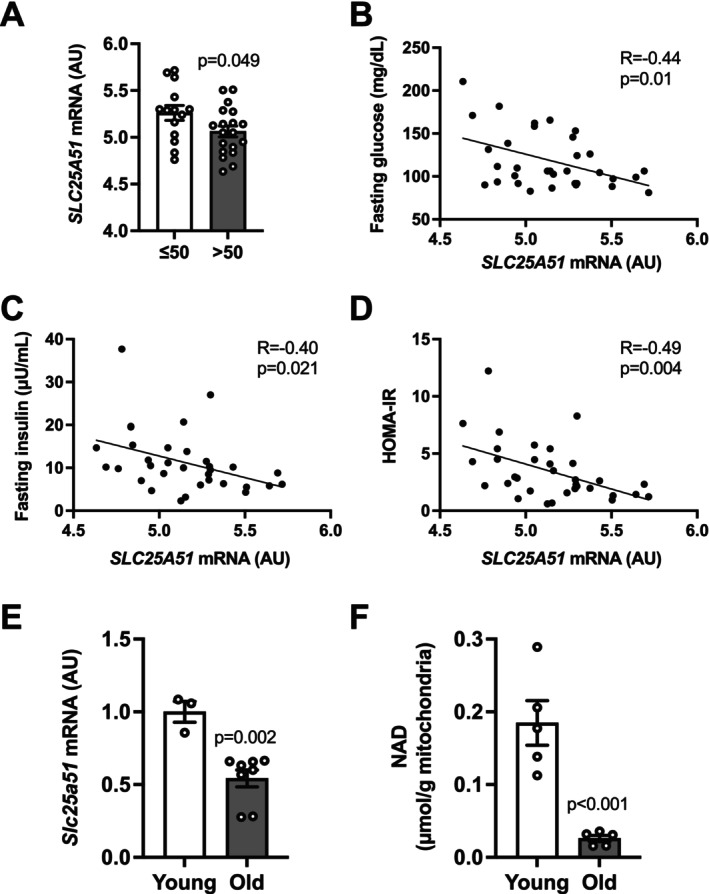
Aging was associated with decreased SLC25A51 expression in adipose tissue. The relationship between subcutaneous adipose tissue SLC25A51 gene expression and age (A) and markers of insulin sensitivity including fasting glucose (B) and insulin (C) concentrations and homeostasis model assessment of insulin resistance (HOMA‐IR) (D) in humans. The gene expression of *Slc25a51* (E) and mitochondrial NAD concentration (F) in inguinal white adipose tissue (iWAT) of young (≤ 4‐month‐old) and old (≥ 22‐month‐old) B6 mice (*n* = 3–8 per group). Values are means ± SEM. Data were analyzed by Student's unpaired *t*‐test (A, E, F). Pearson's correlation coefficient (R) and *p* values are provided (B–D).

We first analyzed the publicly available microarray dataset (GSE27949) obtained from human subcutaneous adipose tissue (Keller et al. 2011). We found that aging was associated with a decrease in adipose tissue *SLC25A51* gene expression in humans (Figure 1A). In addition, adipose tissue *SLC25A51* expression negatively correlated with surrogate markers of insulin sensitivity, such as fasting glucose and insulin concentrations and homeostasis model assessment of insulin resistance (HOMA‐IR) (Figure 1B–D). Consistent with human data, old (≥ 18‐month‐old) B6 mice showed marked decreases in *Slc25a51* gene expression and mitochondrial NAD concentration in inguinal WAT (iWAT), compared with young mice (≤ 4‐month‐old) (Figure 1E,F). These findings led us to hypothesize that defective adipose tissue mitochondrial NAD metabolism could contribute to the development of systemic metabolic complications during aging.

### 3.2 Generation of Adipocyte‐Specific Slc25a51 Knockout (ASKO) Mice

**FIGURE 2:**
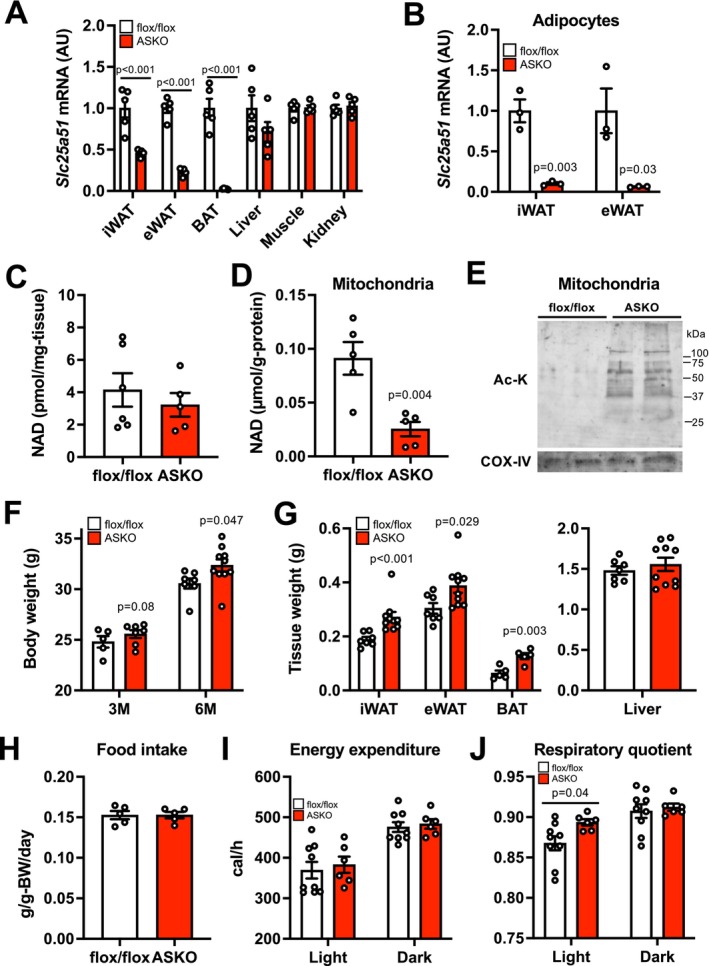
Adipocyte‐specific loss of *Slc25a51* increased fat mass. (A) The gene expression of *Slc25a51* in iWAT, epididymal white adipose tissue (eWAT), brown adipose tissue (BAT), and other organs (*n* = 5 per group). (B) *Slc25a51* gene expression in adipocytes isolated from iWAT and eWAT (*n* = 3 per group). Whole tissue (C) and mitochondrial NAD concentrations (D) in iWAT (*n* = 5 per group). (E) Western blot analysis of acetylated lysine (Ac‐K) and cytochrome c oxidase (COX) IV in iWAT mitochondria. Body weight (F), adipose tissue and liver weights (G) in young adult (3–6 months of age) male mice (*n* = 5–9 per group). (H) Daily food intake (*n* = 5 per group). Energy expenditure (I) and respiratory quotient (J) determined by the indirect calorimetry (*n* = 6–9 per group). Values are means ± SEM. Data were analyzed by Student's unpaired *t*‐test.

To test this hypothesis, we generated and characterized adipocyte‐specific *Slc25a51* knockout (ASKO) mice by crossing the *Adipoq*‐Cre transgenic mice with floxed *Slc25a51* (flox/flox) mice (Figure S1A). In ASKO mice, *Slc25a51* gene expression was markedly reduced in iWAT, epididymal WAT (eWAT), and brown adipose tissue (BAT), but not in other tissues (Figure 2A) while *Slc25a52* and other NAD biosynthetic enzymes were unaffected (Figure S1B,C). *Slc25a51* gene expression was almost completely abolished in adipocytes isolated from iWAT and eWAT of ASKO mice (Figure 2B). Although *Slc25a51* deletion did not affect whole tissue NAD concentration (Figure 2C), it markedly decreased mitochondrial NAD concentration and increased mitochondrial protein lysine acetylation in iWAT (Figure 2D,E).

### 3.3 Adipocyte‐Specific Loss of Slc25a51 Impaired Glucose and Energy Metabolism

**FIGURE 3:**
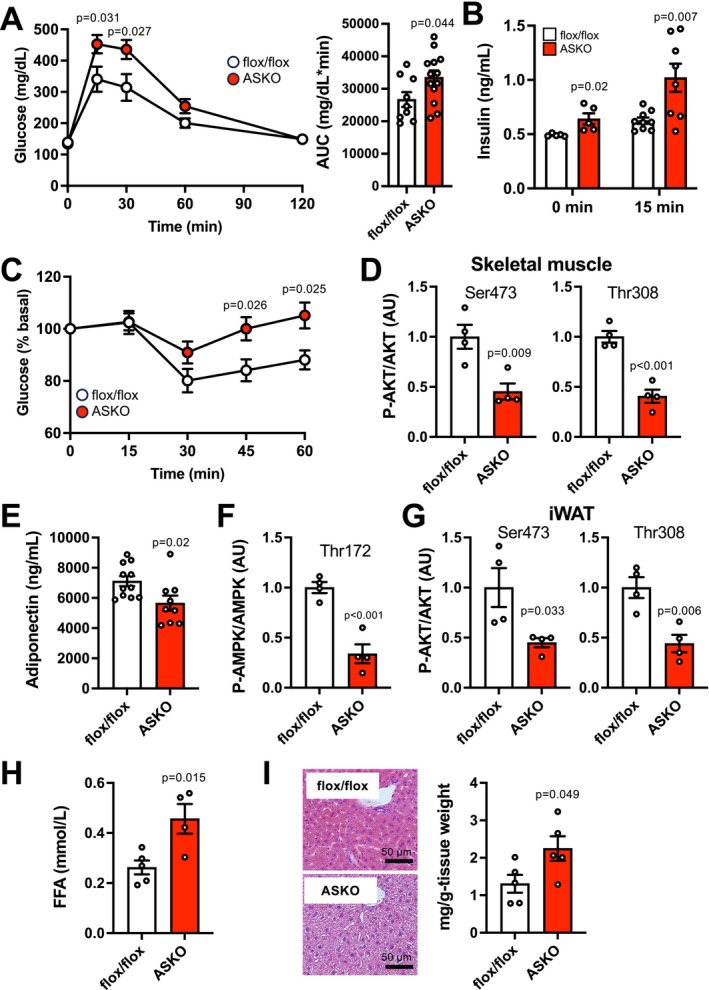
ASKO mice had impaired glucose metabolism. Glucose metabolism in young adult (2–6 months of age) male mice. Blood glucose (A) and plasma insulin concentrations (B) during intraperitoneal glucose tolerance tests (IPGTTs) (*n* = 5–13 per group). (C) Blood glucose concentration during insulin tolerance tests (ITTs) (*n* = 8–13 per group). (D) Insulin‐stimulated AKT phosphorylation at serine 473 (Ser473) and threonine 308 (Thr308) in skeletal muscle (*n* = 4 per group). (E) Plasma adiponectin concentration (*n* = 9–11 per group). (F) AMPK phosphorylation at threonine 172 (Thr172) in skeletal muscle (*n* = 4 per group). (G) Insulin‐stimulated AKT phosphorylation in iWAT (*n* = 4 per group). Plasma free fatty acids (FFA) concentration (H) and hepatic triglyceride content (I) (*n* = 4–5 per group). Values are means ± SEM. Data were analyzed by Student's unpaired *t*‐test.

Adipocyte‐specific loss of *Slc25a51* tended to increase body weight in 3‐month‐old mice, and the difference between genotypes became more evident and significant as mice aged (Figure 2F). Consistent with these findings, ASKO mice had a significant increase in tissue weight of adipose tissue depots, but not liver, compared with flox/flox mice, indicating the development of obesity phenotype in ASKO mice (Figure 2G). There was no difference in food intake and energy expenditure between genotypes (Figure 2H,I, Figure S1D). However, the RQ value, an indicator of relative substrate utilization, was higher during the light period and had less variability in ASKO mice than in flox/flox mice, indicating the development of metabolic inflexibility (Figure 2J). Adipocyte‐specific loss of *Slc25a51* markedly impaired glucose tolerance and increased fasting and glucose‐stimulated plasma insulin concentrations during the IPGTTs in male mice (Figure 3A,B). ASKO mice also had poor responses of glucose disposal to insulin administration (Figure 3C). Consistent with in vivo findings, insulin‐mediated AKT phosphorylation at both serine 473 and threonine 308 was significantly decreased in skeletal muscle obtained from ASKO mice, compared with flox/flox mice (Figure 3D, Figure S2A). ASKO mice had decreases in plasma concentration of adiponectin, a key insulin‐sensitizing adipokine, and in its downstream target regulating glucose uptake, AMPK phosphorylation in skeletal muscle (Figure 3E,F, Figure S2B). Loss of *Slc25a51* decreased insulin‐stimulated AKT phosphorylation in iWAT (Figure 3G, Figure S2C) while it increased plasma FFA and hepatic TG concentrations (Figure 3H,I). Adipocyte‐specific *Slc25a51* deletion similarly caused glucose intolerance, insulin resistance, and hypoadiponectinemia in 13 to 20‐month‐old female mice (Figure S3). Taken together, these findings suggest that adipocyte‐specific loss of *Slc25a51* causes systemic age‐associated metabolic complication.

### 3.4 Loss of Slc25a51 Impaired Mitochondrial Function and Fatty Acid Oxidation in Adipose Tissue

**FIGURE 4:**
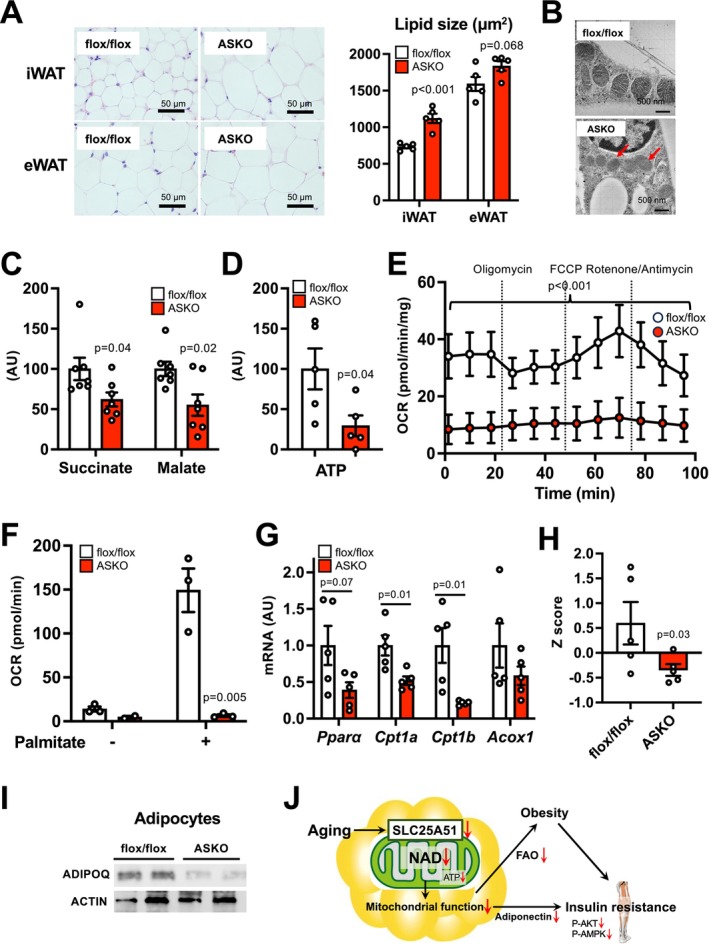
Loss of *Slc25a51* caused severe mitochondrial dysfunction in adipose tissue. (A) Hematoxylin and eosin‐stained sections and quantification of lipid sizes (*n* = 5 per group). (B) An electron microscopical study in iWAT. Key metabolites in TCA cycle (C) and ATP (D) were measured in iWAT. (E) Ex vivo respiratory function determined by the Seahorse system (*n* = 3 per group). Oxygen consumption rates (OCR) were measured in response to oligomycin, carbonyl cyanide p‐trifluoromethoxyphenylhydrazone (FCCP), and rotenone/antimycin. (F) OCR responses to palmitate stimulation (*n* = 3 per group). Gene expression (G) and composite expression values (H) of key FAO regulators in iWAT (*n* = 5 per group). (I) Adiponectin protein expression in adipocytes isolated from iWAT. (J) A schematic overview of the proposed SLC25A51‐mediated mechanism responsible for age‐associated obesity and insulin resistance. Values are means ± SEM. Data were analyzed by Student's unpaired *t*‐test. *Pparα*, peroxisome proliferator‐activated receptor alpha; *Cpt1a*, carnitine palmitoyl transferase 1A; *Cpt1b*, carnitine palmitoyl transferase 1B; *Acox1*; acyl‐coenzyme A oxidase 1.

Consistent with obesity phenotype in vivo, ASKO mice had an increase in lipid droplet size of WAT (Figure 4A). ASKO mice exhibited less dense mitochondrial cristae (Figure 4B) without affecting mitochondrial DNA content (Figure S4A). In addition, ASKO mice had reduced levels of succinate and malate, key mitochondrial TCA cycle metabolites, as well as ATP in iWAT (Figure 4C,D). Ex vivo flux analyzer revealed that *Slc25a51* deletion markedly decreased the mitochondrial oxygen consumption rates (OCR) in iWAT (Figure 4E). In addition, the OCR response to palmitate was substantially reduced in ASKO mice (Figure 4F), suggesting loss of *Slc25a51* led to the reduced capacity for fatty acid oxidation (FAO). *Slc25a51* deletion decreased gene expression of key FAO regulators such as peroxisome proliferator‐activated receptor alpha (*Pparα*), carnitine palmitoyl transferase 1A (*Cpt1a*), and *Cpt1b* in iWAT (Figure 4G,H), while it did not affect protein expression of the subunits of electron transport chain (ETC) complex (Figure S4B). ASKO mice also had increases in the levels of several pentose phosphate pathway metabolites involved in regulating *de novo* lipogenesis (Horecker 2002) and gene expression of mitochondrial stress markers such as growth differentiation factor 15 (GDF15) and fibroblast growth factor 21 (FGF21) (Figure S4C,D). Loss of *Slc25a51* overall did not affect gene expression of peroxisome proliferator‐activated receptor gamma (PPARG) lipogenic targets and tended to increase acetyl‐CoA carboxylase (*Acaca*) and fatty acid synthase (*Fas*) in iWAT (Figure S4E). Finally, protein expression of adiponectin, which is known to be associated with mitochondrial function (Kusminski and Scherer 2012), was markedly decreased in adipocytes isolated from iWAT of ASKO mice (Figure 4I). In BAT, *Slc25a51* deletion markedly decreased mitochondrial NAD concentration and similarly induced lipid accumulation and whitening phenotype and impaired mitochondrial function and FAO capacity (Figure S5). Taken together, these results suggest that loss of *Slc25a51* causes mitochondrial dysfunction and impaired FAO in adipose tissue, which could contribute to the development of systemic metabolic diseases including obesity, insulin resistance, and hypoadiponectinemia (Figure 4J).

### 3.5 Slc25a51 Overexpression Prevented Obesity and Insulin Resistance Caused by Aging

**FIGURE 5:**
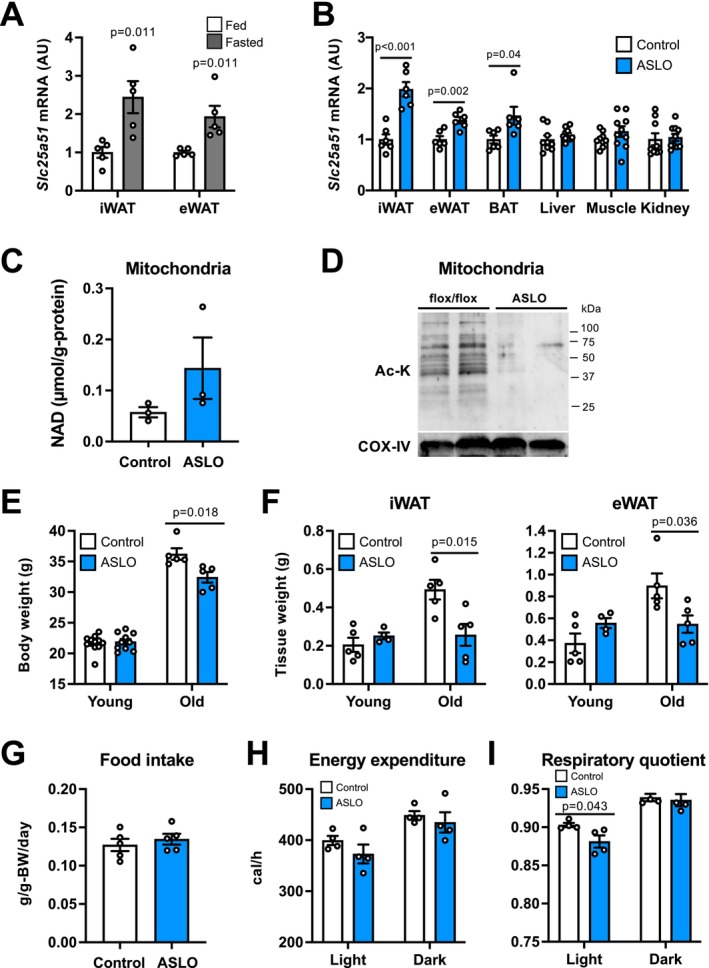
ASLO mice were protected from age‐associated obesity. (A) The gene expression of *Slc25a51* in iWAT and eWAT of fed and 24 h‐fasted B6 mice (*n* = 5 per group). (B) *Slc25a51* gene expression in control and adipocyte‐specific *Slc25a51* overexpressing (ASLO) mice (*n* = 6–10 per group). (C) Mitochondrial NAD concentrations in iWAT of middle‐aged (11 to 14‐month‐old) mice (*n* = 3 per group). (D) Western blot analysis of Ac‐K and COX IV in iWAT mitochondria. Body weight (E) and iWAT and eWAT tissue weights (F) in young (≤ 4‐month‐old) and old (≥ 18‐month‐old) male mice (*n* = 4–11 per group). (G) Daily food intake in old mice (*n* = 5 per group). Energy expenditure (H) and respiratory quotient (I) in old mice (*n* = 4 per group). Values are means ± SEM. Data were analyzed by Student's unpaired *t*‐test.

We next explored the therapeutic potential of adipose tissue mitochondrial NAD biology in age‐associated metabolic diseases. *Slc25a51* gene expression was significantly increased in WAT depots after 24 h‐fasting, which is known to improve whole‐body metabolic function (Figure 5A). Based on these results, we hypothesize that increased *Slc25a51* expression could induce metabolic benefits on glucose and energy metabolism. To test the hypothesis, we generated adipocyte‐specific *Slc25a51* overexpressing (ASLO) mice using the murine *Adipoq* promoter (Wang et al. 2010). *Slc25a51* expression was moderately increased in adipose tissue depots, but not in other organs (Figure 5B). Th increase was quantitatively similar to that induced by fasting. *Slc25a52* and other NAD biosynthetic enzymes were unaffected (Figure S6A,B). *Slc25a51* overexpression tended to increase mitochondrial NAD concentration while it decreased mitochondrial protein lysine acetylation in iWAT of middle‐aged (11 to 14‐month‐old) mice (Figure 5C,D). Adipocyte‐specific *Slc25a51* activation significantly decreased body weight in old mice but not in young male mice (Figure 5E). Old ASLO mice had a significant decrease in tissue weight of WAT depots, compared with flox/flox mice (Figure 5F), indicating *Slc25a51* overexpression in adipocytes prevented age‐associated obesity. There was no difference in food intake and energy expenditure between genotypes in old mice (Figure 5G,H, Figure S6C). The RQ value was lower during the light period in old ASLO mice than in flox/flox mice (Figure 5I).

**FIGURE 6:**
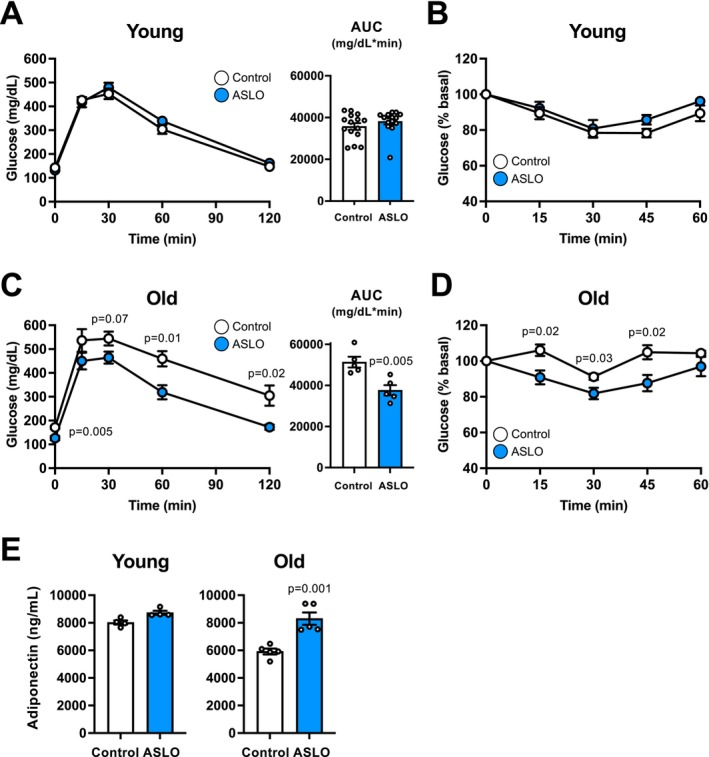
Adipocyte‐specific activation of SLC25A51 improved glucose metabolism and insulin sensitivity in aged mice. Glucose metabolism was evaluated in young (≤ 4‐month‐old) and old (≥ 18‐month‐old) male mice (*n* = 5–15 per group). Blood glucose concentrations during the IPGTTs (A, C) and ITTs (B, D) in young (A, B) and old (C, D) mice (*n* = 5 per group). (E) Plasma adiponectin concentrations. Values are means ± SEM. Data were analyzed by Student's unpaired *t*‐test.

Old (≥ 18‐month‐old) male ASLO mice, but not young (≤ 4‐month‐old) ASLO mice, exhibited improved glucose tolerance, enhanced insulin sensitivity, and increased plasma adiponectin concentration, compared with their control mice (Figure 6). In addition, old (19 to 20‐month‐old) female ASLO mice displayed similar metabolic improvements (Figure S7). Taken together, these findings suggest that adipocyte‐specific *Slc25a51* activation prevented age‐associated obesity and insulin resistance.

## 4 Discussion

In the present study, we found that defective mitochondrial NAD metabolism impairs mitochondrial function and FAO capacity in adipose tissue, leading to the development of age‐associated systemic metabolic abnormalities including obesity, glucose intolerance, insulin resistance, hyperinsulinemia, metabolic inflexibility, dyslipidemia, and hepatosteatosis. In addition, we revealed the novel relationship among adipose tissue *SLC25A51* expression, aging, and insulin sensitivity in humans. These findings reinforce the pathophysiological and clinical significance of adipose tissue NAD biology in regulating whole‐body energy metabolism.

Our results demonstrate the importance of SLC25A51 in regulating adipose tissue mitochondrial NAD homeostasis, which is consistent with data obtained from the previous studies conducted in vitro (Girardi et al. 2020; Kory et al. 2020; Luongo et al. 2020). We recently generated and metabolically characterized mice with adipocyte‐specific deletion of nicotinamide phosphoribosyltransferase (NAMPT) or nicotinamide mononucleotide adenylyltransferase 1 (NMNAT1). NAMPT is a rate‐limiting enzyme in the salvage pathway which generates NMN from nicotinamide in the cytosol and it is supposed to regulate NAD concentrations in multiple subcellular components including mitochondria and nucleus through the distribution of NMN and NAD (Stein and Imai 2012; Yoshino et al. 2018). NMNAT1 is localized to the nucleus and it synthesizes NAD from NMN governing NAD homeostasis in the nucleus (Iqbal et al. 2022; Yamaguchi et al. 2023). The metabolic phenotypes observed in ASKO mice are similar to those in adipocyte‐specific *Nampt* knockout (ANKO) mice we have recently reported (Franczyk et al. 2021; Nielsen et al. 2018; Yamaguchi et al. 2019). However, obesity phenotype is not evident in ANKO mice. In addition, loss of NAMPT, but not SLC25A51, decreased mitochondrial DNA content in iWAT (Franczyk et al. 2021; Yamaguchi et al. 2019). The mechanisms responsible for these apparently inconsistent findings remain unclear but could be explained by the hypothesis that NAMPT deficiency decreases NAD concentrations in multiple subcellular components including the nucleus, which in turn affects the lipogenic capacity and mitochondrial biogenesis in adipose tissue. Supporting our hypothesis, we found that loss of NAMPT decreases PPARG activity likely through inhibition of SIRT1 deacetylase activity and reduces expression of PPARG lipogenic targets particularly in aged mice (Qi et al. 2024; Stromsdorfer et al. 2016), indicating nuclear NAD depletion and subsequent PPARG inactivation could confound the effects of defective mitochondrial NAD metabolism on lipid accumulation in ANKO mice. In contrast to ASKO and ANKO mice, adipocyte‐specific NMNAT1 knockout (ANMT1KO) mice have normal adipose tissue and whole‐body energy metabolism despite a reduction in nuclear NAD concentration in adipose tissue (Yamaguchi et al. 2023). Taken together, the results obtained from the studies conducted in three distinct knockout mouse models indicate the complex subcellular component‐specific role of NAD biology in regulating metabolic function. Given that ASKO mice had normal whole tissue NAD concentration, we can speculate that *Slc25a51* inhibition could lead to a redistribution of NAD subcellular pool and increase NAD concentrations in other subcellular components, which also contributes to the pathogenesis of metabolic abnormalities observed in ASKO mice. Indeed, recent data obtained from the studies conducted in cancer cell lines such as U2OS and MOLM‐13 found that knockdown of *Slc25a51* elevates cytosolic and nuclear NAD concentrations (Guldenpfennig et al. 2023; Lu et al. 2024). Future studies are warranted to further investigate the subcellular component‐specific role of NAD biology and understand the regulatory mechanism responsible for the redistribution of NAD subcellular pool during aging.

Recent studies show that genetic ablation of nicotinamide mononucleotide adenylyltransferase 3 (NMNAT3), a NAD biosynthetic enzyme localized in mitochondria (Lau et al. 2009), does not affect mitochondrial NAD metabolism in vivo (Yamamoto et al. 2016). It is therefore proposed that NAMPT produces NAD from NMN predominantly in cytosol and synthesized NAD is transported into mitochondria through SLC25A51 (Kory et al. 2020; Luongo et al. 2020). Given that adipose tissue NAMPT expression decreases with aging (Yoshino et al. 2011), these findings suggest that decreases in cytosolic NAMPT and mitochondrial SLC25A51 expression synergistically contribute to age‐associated loss of mitochondrial NAD concentration in adipose tissue. In addition, activation of NAD consuming enzyme(s) could affect mitochondrial NAD metabolism during aging (Camacho‐Pereira et al. 2016; Chini et al. 2023; Chini et al. 2021; Rajman et al. 2018). Indeed, data obtained from the recent studies conducted in CD38 deficient mice suggest that CD38 is an important mediator for age‐associated NAD decline and mitochondrial dysfunction (Camacho‐Pereira et al. 2016). Additional studies are required to investigate the complex regulatory mechanism of age‐associated alterations in mitochondrial NAD metabolism in adipose tissue.

The precise mechanisms that link defective mitochondrial NAD metabolism and obesity phenotype observed in ASKO mice remain unclear but could involve impaired mitochondrial FAO and oxidative phosphorylation in adipose tissue. Previous studies have demonstrated the close relationship between reduced FAO capacity and obesity (Soro‐Arnaiz et al. 2016). For example, adipocyte‐specific inactivation of the mitochondrial transcription factor A (TFAM) increases palmitate oxidation in adipose tissue and protects mice from age‐induced obesity (Vernochet et al. 2012). Genetic inhibition of the key mitochondrial regulator, such as Cox5b or paraoxonase‐3 (PON3), decreases FAO in adipocytes while it increases the lipid droplet size (Shih et al. 2015; Soro‐Arnaiz et al. 2016). Because aging is associated with reduced FAO capacity in adipose tissue (Soro‐Arnaiz et al. 2016), our results suggest that impaired FAO caused by SLC25A51 inactivation is likely involved in the mechanisms of age‐associated obesity. In addition, we found that *Slc25a51* deletion increases mitochondrial protein lysine acetylation, indicating deactivation of mitochondrial sirtuin(s), and it decreases gene expression of PPARα and its targets involved in regulating FAO, such as *Cpt1a* and *Cpt1b*. Although we reported adipocyte‐specific loss of SIRT3, a key mitochondrial sirtuin regulating acetylation and activities of various FAO enzymes (van de Ven et al. 2017), does not affect whole‐body energy metabolism (Porter et al. 2018), we cannot exclude the possibility that SIRT3 is involved in the mechanism regulating FAO through modulation of the metabolic pathways that we did not evaluate in the past study. Indeed, it was recently reported that SIRT3 regulates PPARα activity, *Cpt1b* expression and acylcarnitine metabolism in adipose tissue (Zhang et al. 2024). Obesity leads to the development of systemic metabolic complications, particularly multi‐organ insulin resistance, in ASKO mice. In addition, hypoadiponectinemia could contribute to the development of insulin resistance in ASKO mice. Indeed, previous studies found mitochondrial dysfunction, manifested by impaired FAO, links to decreases in production and release of adiponectin (Koh et al. 2007). Thus, hypoadiponectinemia is caused, at least in part, by adipose tissue mitochondrial dysfunction. Taken together, these mutually unexclusive mechanisms could link defective mitochondrial NAD metabolism in adipose tissue with obesity and insulin resistance.

We found that moderate *Slc25a51* overexpression tends to increase mitochondrial NAD concentration and decreases mitochondrial protein lysine acetylation in iWAT of middle‐aged mice. Given that aging is associated with decreases in *Slc25a51* expression and mitochondrial NAD concentration, our results indicate that *Slc25a51* overexpression prevents age‐associated decline in mitochondrial NAD concentration and sirtuin(s) activity and thus protects against age‐associated obesity and insulin resistance. Because an increase in *Slc25a51* expression is equivalent to that induced by fasting, SLC25A51 could mediate fasting‐induced beneficial metabolic effects including improved glucose tolerance and insulin sensitivity. Our results are consistent with data obtained from the recent studies that found NMNAT3 overexpression increases mitochondrial NAD concentration and FAO and ameliorates age‐associated insulin resistance (Gulshan et al. 2018). In addition, NAD boosters, such as NMN, nicotinamide riboside (NR), CD38 inhibitor, and poly (ADP‐ribose) polymerases (PARPs) inhibitors, effectively enhance mitochondrial function and improve glucose metabolism and insulin sensitivity in aged mice (Chini et al. 2023; Katsyuba et al. 2020; Rajman et al. 2018; Yoshino et al. 2018). Taken together, these findings revealed the significance of mitochondrial NAD biology as an attractive therapeutic target for adipose tissue mitochondrial dysfunction and systemic metabolic abnormalities caused by aging.

The present study has several limitations. First, we were unable to measure NAD concentrations in other subcellular components such as the nucleus and cytosol due to the technical limitation. Thus, future studies are needed to improve the sensitivity for the accurate measurements of subcellular NAD metabolites in WAT. Second, the molecular mechanisms explaining the difference in obesity phenotype between ASKO and ANKO mice are not yet fully elucidated. Third, although we did not find any change in energy expenditure or food intake in ASKO or ASLO mice, we cannot rule out the possibility that the methods used in the present study are not sensitive enough to detect the small changes that accumulate over time during aging. Finally, the causal relationship among adipose tissue *SLC25A51* expression, aging, and insulin resistance in humans remains unclear, which is a common limitation in human studies, and it could be affected by potential confounders such as body mass index (BMI), sex, and comorbidities. Nonetheless, our results could provide new clinical insight into adipose tissue mitochondrial NAD biology in human pathophysiology.

In conclusion, the results from the present study illustrate the critical role and therapeutic potential of adipocyte mitochondrial NAD transporter SLC25A51 in age‐associated metabolic diseases. Recent clinical studies found that NMN supplementation stimulates NAD biosynthesis and improves cardiometabolic function, such as insulin sensitivity, blood pressure, and lipid profile in humans (Pencina et al. 2023; Yoshino et al. 2021). Additional studies are required to further explore the translational potential of the results from the present study to clinical practice and to determine whether adipocyte mitochondrial NAD biology is a new therapeutic target for combating age‐associated metabolic diseases in humans.

## Author Contributions

J.Y. conceptualized and designed the project. D.K., K.Y., S.K., R.M., K.K. Y.K., S.M., M.K, A.S., S.T., T.A., K.K., A.H., S.Y., and J.Y. performed the experiments and analyzed the data. H.I., T.K., T.Y., T.N., K.H., and J.Y. provided intellectual input for the study design and data interpretation. All authors reviewed and edited the manuscript. J.Y. is the guarantor of this work and, as such, had full access to all the data in the study and takes responsibility for the integrity of the data and the accuracy of the data analysis.

## Funding

This work was supported by the JSPS Kakenhi (20K23382, 24K02506, 25K02627, 21K18270), AMED‐PRIME (25gm6710007h0004), and the grants from the Naito Foundation, Novo Nordisk Pharma Ltd., Astellas Foundation for Research on Metabolic Disorders, Japan Geriatrics Society Research Grant Award in Geriatrics and Gerontology, Salt Science Research Foundation, and Keio University Program for the Advancement of Next Generation Research Projects.

## Conflicts of Interest

D.K., K.Y., S.K., R.M., K.K., Y.K., A.H., S.M., A.K., S.Y., T.A., S.Y., H.I., T.K., T.N., and K.H. had no potential conflicts of interest relevant to this article. M.K.was employed by a donation‐funded division supported by the Nexera Pharma Japan Co. Ltd., Toa Eiyo Ltd., Nippon Boehringer Ingelheim Co. Ltd., Novo Nordisk Pharma Ltd., Takara Bio Inc., and BioStream Inc. K.K. received the grants from the JSPS Kakenhi, Salt Science Research Foundation, and Keio University Program for the Advancement of Next Generation Research Projects. T.Y. received grants or contract from Novo Nordisk Pharma Ltd., Mitsubishi Tanabe Pharma Corporation, Takeda Pharmaceutical Company Limited., Ono Pharmaceutical Co. Ltd., Sumitomo Pharma Co. Ltd., Nipro Corporation, MED MIRAI Inc., Boehringer Ingelheim GmbH Japan, Kowa Pharmaceutical company, limited., NITTO BOSEKI CO. LTD., Asahi Mutual Life Insurance Company, SANWA KAGAKU KENKYUSHO CO. LTD., and received payment or honoraria from Abbott Japan LLC, Daiichi Sankyo Company, Limited, Sanofi K.K., Viatris Inc. Medtronic Japan, Astellas Pharma Inc., Astra Zeneca K.K., TERUMO CORPORATION, Taisho Pharmaceutical Co. Ltd., Amgen inc., ARKRAY Marketing Inc., Bayer Yakuhin Ltd., Lindsay Advice Co. Ltd., Kyowa Kirin Co. Ltd., SANWA KAGAKU KENKYUSHO CO. LTD., GSK, Ono Pharmaceutical Co. Ltd., Merck Sharp & Dohme Co.(Merck & Co.), Sumitomo Pharma Co. Ltd., TEIJIN PHARMA LIMITED., Boehringer Ingelheim GmbH Japan, Novo Nordisk Pharma Ltd., Eli Lilly and Company, Kowa Pharmaceutical company, limited., Nipro Corporation, Mitsubishi Tanabe Pharma Corporation, Amgen inc. Dexcom Japan, NITTO BOSEKI CO. LTD., View Send ICT Co. Ltd. J.Y. is listed as an inventor on patent applications related to NMN and adiponectin (US20180228824, JP2018131418A), and received the grants from the JSPS Kakenhi, Naito Foundation, Novo Nordisk Pharma Ltd., Astellas Foundation for Research on Metabolic Disorders, and Japan Geriatrics Society (JGS) Research Grant Award in Geriatrics and Gerontology. J.Y.'s family received stock or stock options from Eli Lilly.

## Supporting information


**Figure S1:** Adipose tissue gene expression of enzymes involved in NAD metabolism and energy expenditure in flox/flox and adipocyte‐specific Slc25a51 knockout (ASKO) mice. (A) Generation of floxed‐Slc25a51 (flox/flox) mice. (B) Slc25a52 expression in adipose tissue depots (*n* = 5–6 per group). (C) Gene expression of NAD biosynthetic enzymes in inguinal white adipose tissue (iWAT) and epididymal white adipose tissue (eWAT) (*n* = 5–6 per group). (D) Energy expenditure (EE) was determined by the indirect calorimetry (*n* = 6–9 per group). EE values were adjusted for body mass by Analysis of Covariance (ANCOVA). Values are means ± SEM. Data were analyzed by Student's unpaired *t*‐test.
**Figure S2:** Western blot images of AKT and AMPK phosphorylation Western blot images of insulin‐stimulated AKT phosphorylation at serine 473 (Ser473) and threonine 308 (Thr308) (A) and AMPK phosphorylation at threonine 172 (Thr172) (B) in skeletal muscle obtained from young adult (2–6 months of age) male mice. (C) Western blot images of insulin‐stimulated AKT phosphorylation at serine 473 (Ser473) and threonine 308 (Thr308) in iWAT obtained from young adult (2–6 months of age) male mice.
**Figure S3:** Metabolic phenotype in female flox/flox and ASKO mice Glucose metabolism was evaluated in 13 to 20‐month‐old female mice. Blood glucose (A) and fasted plasma insulin concentrations (B) during intraperitoneal glucose tolerance tests (IPGTTs). (C) Blood glucose concentration during insulin tolerance tests (ITTs) (*n* = 4–7 per group). (D) Plasma adiponectin concentration (*n* = 4–7 per group). Values are means ± SEM. Data were analyzed by Student's unpaired *t*‐test.
**Figure S4:** Loss of SLC25A51 induced mitochondrial dysfunction and stress in adipose tissue (A) Mitochondrial DNA content in iWAT (*n* = 4 per group). (B) Western blot analysis of the subunits of electron transport chain (ETC) complex in iWAT. (C) Metabolites in pentose phosphate pathway in iWAT (*n* = 7 per group). R5P; ribose‐5‐phosphate, E4P; erythrose‐4‐phosphate, S7P; sedoheptulose‐7‐phosphate, DHAP; dihydroxyacetone phosphate. (D) Gene expression of mitochondrial stress markers, growth differentiation factor 15 (GDF15) and fibroblast growth factor 21 (FGF21), in iWAT (*n* = 5 per group). (E) Gene expression of peroxisome proliferator‐activated receptor gamma (PPARG) lipogenic targets in iWAT (*n* = 5 per group). Values are means ± SEM. Data were analyzed by Student's unpaired *t*‐test. Pparγ, peroxisome proliferator‐activated receptor gamma; Acaca, acetyl‐CoA carboxylase; Fas, fatty acid synthase; Scd1, stearoyl‐CoA desaturase 1; Fabp4; fatty acid binding protein 4; Cd36, cluster of differentiation 36; Glut4, glucose transporter 4.
**Figure S5:** The effects of Slc25a51 deletion on mitochondrial function in brown adipose tissue (BAT). (A) Mitochondrial NAD concentrations in BAT (*n* = 6 per group). (B) Hematoxylin and eosin‐stained sections and quantification of lipid sizes (*n* = 5 per group). (C) An electron microscopical study in BAT. (D) Mitochondrial DNA content in BAT (*n* = 5 per group). Key metabolites in TCA cycle (E) and ATP (F) were measured in BAT (*n* = 3–7 per group). (G) Ex vivo respiratory function was determined by the Seahorse system (*n* = 3 per group). Oxygen consumption rates (OCR) at the baseline and responses to palmitate stimulation. Values are means ± SEM. Data were analyzed by Student's unpaired *t*‐test.
**Figure S6:** Adipose tissue gene expression of enzymes involved in NAD metabolism and energy expenditure in flox/flox and adipocyte‐specific Slc25a51 overexpressing (ASLO) mice (A) Slc25a52 expression in inguinal white adipose tissue (iWAT), epididymal white adipose tissue (eWAT), and brown adipose tissue (BAT) (*n* = 6 per group). (B) Gene expression of NAD biosynthetic enzymes in iWAT and eWAT (*n* = 6 per group). (C) Energy expenditure (EE) was determined by the indirect calorimetry (*n* = 4 per group). EE values were adjusted for body mass by Analysis of Covariance (ANCOVA). Values are means ± SEM. Data were analyzed by Student's unpaired *t*‐test.
**Figure S7:** Metabolic phenotype in old female flox/flox and ASLO mice Glucose metabolism was evaluated in old (19 to 20‐month‐old) female mice. Blood glucose concentrations during intraperitoneal glucose tolerance tests (IPGTTs) (A) and insulin tolerance tests (ITTs) (B) (*n* = 4–6 per group). (C) Plasma adiponectin concentration (*n* = 4–6 per group). Values are means ± SEM. Data were analyzed by Student's unpaired *t*‐test.
**Table S1:** Assay kits.
**Table S2:** Sequence of primers for real‐time PCR.
**Table S3:** Antibodies.

## Data Availability

Data and resources are available on reasonable request.
