# Supplementary material for: The Mitochondrial NAD Transporter SLC25A51 in Adipocytes Regulates Adipose Tissue Mitochondrial Function and Systemic Metabolism During Aging
Source: Aging Cell. 2026 Apr 21;25(5):e70509. doi: 10.1111/acel.70509 (PMC13100308; doi:10.1111/acel.70509)
Supplement: Supplementary file 1 — Figure S1: Adipose tissue gene expression of enzymes involved in NAD metabolism and energy expenditure in flox/flox and adipocyte‐specific Slc25a51 knockout (ASKO) mice. (A) Generation of floxed‐Slc25a51 (flox/flox) mice. (B) Slc25a52 expression in adipose tissue depots (n = 5–6 per group). (C) Gene expression of NAD biosynthetic enzymes in inguinal white adipose tissue (iWAT) and epididymal white adipose tissue (eWAT) (n = 5–6 per group). (D) Energy expenditure (EE) was determined by the indirect calorimetry (n = 6–9 per group). EE values were adjusted for body mass by Analysis of Covariance (ANCOVA). Values are means ± SEM. Data were analyzed by Student's unpaired t‐test. Figure S2: Western blot images of AKT and AMPK phosphorylation Western blot images of insulin‐stimulated AKT phosphorylation at serine 473 (Ser473) and threonine 308 (Thr308) (A) and AMPK phosphorylation at threonine 172 (Thr172) (B) in skeletal muscle obtained from young adult (2–6 months of age) male mice. (C) Western blot images of insulin‐stimulated AKT phosphorylation at serine 473 (Ser473) and threonine 308 (Thr308) in iWAT obtained from young adult (2–6 months of age) male mice. Figure S3: Metabolic phenotype in female flox/flox and ASKO mice Glucose metabolism was evaluated in 13 to 20‐month‐old female mice. Blood glucose (A) and fasted plasma insulin concentrations (B) during intraperitoneal glucose tolerance tests (IPGTTs). (C) Blood glucose concentration during insulin tolerance tests (ITTs) (n = 4–7 per group). (D) Plasma adiponectin concentration (n = 4–7 per group). Values are means ± SEM. Data were analyzed by Student's unpaired t‐test. Figure S4: Loss of SLC25A51 induced mitochondrial dysfunction and stress in adipose tissue (A) Mitochondrial DNA content in iWAT (n = 4 per group). (B) Western blot analysis of the subunits of electron transport chain (ETC) complex in iWAT. (C) Metabolites in pentose phosphate pathway in iWAT (n = 7 per group). R5P; ribose‐5‐phosphate, E4P; erythros [file ACEL-25-e70509-s001.pdf]

Supplemental Table 1. Assay kits

Assay kit	Source	Identifier
Plasma insulin ELISA kits	Morinaga Institute of Biological Science	#M1104
Plasma adiponectin ELISA kits	R&D systems	MRP300
Plasma free fatty acids (FFA) assay kits	FUJIFILM Wako Pure Chemical Corporation	#299-94301
Plasma hepatic triglyceride (TG) assay kits	FUJIFILM Wako Pure Chemical Corporation	#291-94,501

Supplemental Table 2. Sequence of primers for real-time PCR

Gene	Accession Number	Primer sequences (5'-3') Forward	Reverse
Slc25a51	NM_001009949	ATGATGGACTCCGAAGCACAT	GGGTAAGTGATCGCCACGTT
Slc25a52	N/A	ACACGGTTCGCCGTCGTATGAT	AAAGCCTTGCTCCCTTCATCGC
Nampt	NM_021524	GCAGAAGCCGAGTTCAACATC	TTTTCACGGCATTCAAAGTAGGA
Nmnat1	NM_133435	TGGCTCTTTTAACCCCATCAC	TCTTCTTGTACGCATCACCGA
Nmnat3	NM_144533	CCTGTGGTTCCTTCAACCCC	AGATGATGCCCTCAATCACCT
Naprt1	NM_172607	TGCTCACCGACCTCTATCAGG	CGAAGGAGCCTCCGAAAGG
Nadsyn1	NM_030221	ACGGCTGCTCACTACTTGTTA	CTGAGAACCGAGGCAACTTC
Pparα	NM_011144	AGAGCCCCATCTGTCCTCTC	ACTGGTAGTCTGCAAAACCAAA
Cpt1a	NM_013495	CTCCGCCTGAGCCATGAAG	CACCAGTGATGATGCCATTCT
Cpt1b	NM_009948	GCACACCAGGCAGTAGCTTT	CAGGAGTTGATTCCAGACAGGTA
Acox1	NM_015729	TAACTTCCTCACTCGAAGCCA	AGTTCCATGACCCATCTCTGTC
Gdf15	NM_011819	GAGCTACGGGGTCGCTTC	GGGACCCCAATCTCACCT
Fgf21	NM_020013	CTGCTGGGGGTCTACCAAG	CTGCGCCTACCACTGTTCC
Pparγ	NM_001127330	GGAAGACCACTCGCATTCCTT	GTAATCAGCAACCATTGGGTCA
Acaca	NM_133360	GATGAACCATCTCCGTTGGC	GACCCAATTATGAATCGGGAGTG
Fas	NM_007988	GGAGGTGGTGATAGCCGGTAT	TGGGTAATCCATAGAGCCCAG
Scd1	NM_009127	TTCTTGCGATACACTCTGGTGC	CGGGATTGAATGTTCTTGTCGT
Fabp4	NM_024406	AAGGTGAAGAGCATCATAACCCT	TCACGCCTTTCATAACACATTCC
Cd36	NM_001159558	ATGGGCTGTGATCGGAACTG	TTTGCCACGTCATCTGGGTTT
Glut4	NM_009204	CTGTCGCTGGTTTCTCCAA	CTGCTCTAAAAGGGAAGGTGTC
36b4	NM_007475	GCAGACAACGTGGGCTCCAAGCAGAT	GGTCCTCCTTGGTGAACACGAAGCCC

Slc25a51, solute carrier family 25 member 51; *Slc25a52*, solute carrier family 25 member 52; *Nampt*, nicotinamide phosphoribosyltransferase; *Nmnat1*, nicotinamide mononucleotide adenylyltransferase 1; *Nmnat3*, nicotinamide mononucleotide adenylyltransferase 3; *Naprt1*, nicotinate phosphoribosyltransferase 1; *Nadsyn1*, nicotinamide adenine dinucleotide synthase 1; *Pparα*, peroxisome proliferator-activated receptor alpha; *Cpt1a*, carnitine palmitoyl transferase 1A; *Cpt1b*, carnitine palmitoyl transferase 1B; *Acox1*; acyl-coenzyme A oxidase 1; *Gdf15*, growth differentiation factor 15; *Fgf21*, Fibroblast Growth Factor 21; *Pparγ*, peroxisome proliferator-activated receptor gamma; *Acaca*, acetyl-CoA carboxylase; *Fas*, fatty acid synthase; *Scd1*, stearoyl-CoA desaturase 1; *Fabp4*; fatty acid binding protein 4; *Cd36*, cluster of differentiation 36; *Glut4*, glucose transporter 4; 3*6b4*, acidic ribosomal phosphoprotein P0.

Supplemental Table 3. Antibodies

Antibody	Source	Identifier
rabbit monoclonal anti-phospho-Akt (Ser473) antibody	Cell Signaling Technology	#4060
rabbit monoclonal anti-phospho-Akt (Thr308) antibody	Cell Signaling Technology	#13038
rabbit anti-Akt antibody	Cell Signaling Technology	#9272
rabbit monoclonal phospho-AMPKα (Thr172) antibody	Cell Signaling Technology	#2535
rabbit AMPKα antibody	Cell Signaling Technology	#2532
rabbit monoclonal adiponectin antibody	Cell Signaling Technology	#2789
rabbit monoclonal COX IV (3E11) antibody	Cell Signaling Technology	#4850
rabbit polyclonal acetylated-lysine antibody	Cell Signaling Technology	#9441
mouse monoclonal total OXPHOS rodent antibody cocktail	Abcam	ab110413
rabbit polyclonal alpha-Tubulin antibody	Cell Signaling Technology	#2144
rabbit IgG, HRP-linked Antibody	Cell Signaling Technology	#7074

Supplemental Figure 1

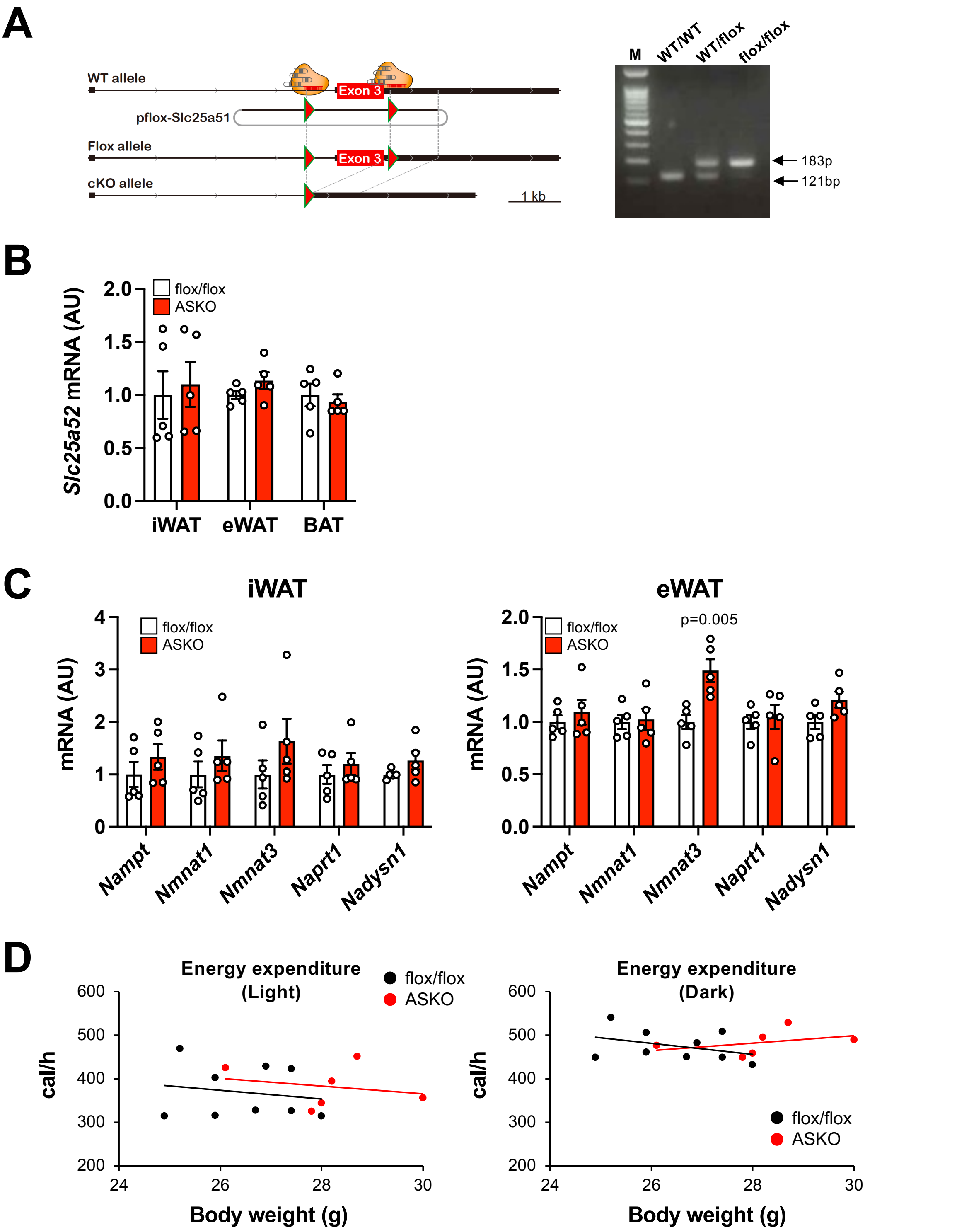

Supplemental Figure 1. Adipose tissue gene expression of enzymes involved in NAD metabolism and energy expenditure in flox/flox and adipocyte-specific *Slc25a51* knockout (ASKO) mice

(**A**) Generation of floxed-*Slc25a51* (flox/flox) mice. (**B**) *Slc25a52* expression in adipose tissue depots (n=5-6 per group). (**C**) Gene expression of NAD biosynthetic enzymes in inguinal white adipose tissue (iWAT) and epididymal white adipose tissue (eWAT) (n=5-6 per group). (**D**) Energy expenditure (EE) was determined by the indirect calorimetry (n=6-9 per group). EE values were adjusted for body mass by Analysis of Covariance (ANCOVA). Values are means ± SEM. Data were analyzed by Student's unpaired t test.

Supplemental Figure 2

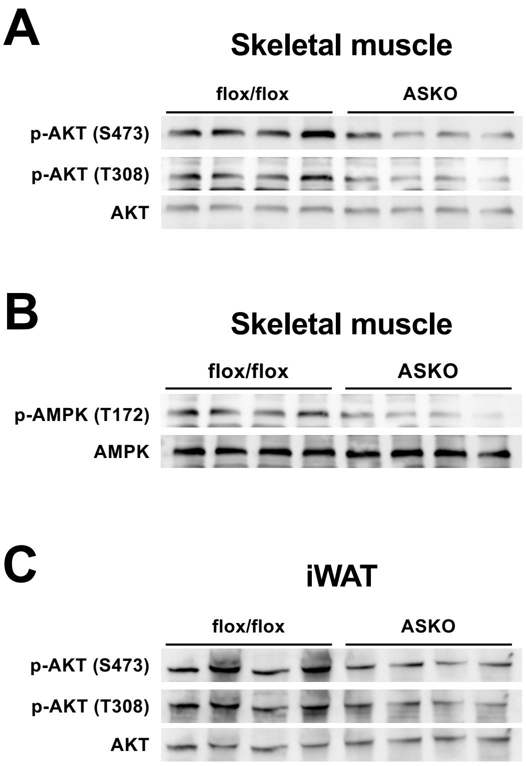

Supplemental Figure 2. Western blot images of AKT and AMPK phosphorylation

Western blot images of insulin-stimulated AKT phosphorylation at serine 473 (Ser473) and threonine 308 (Thr308) (**A**) and AMPK phosphorylation at threonine 172 (Thr172) (**B**) in skeletal muscle obtained from young adult (2-6 months of age) male mice. (**C**) Western blot images of insulin-stimulated AKT phosphorylation at serine 473 (Ser473) and threonine 308 (Thr308) in iWAT obtained from young adult (2-6 months of age) male mice.

Supplemental Figure 3

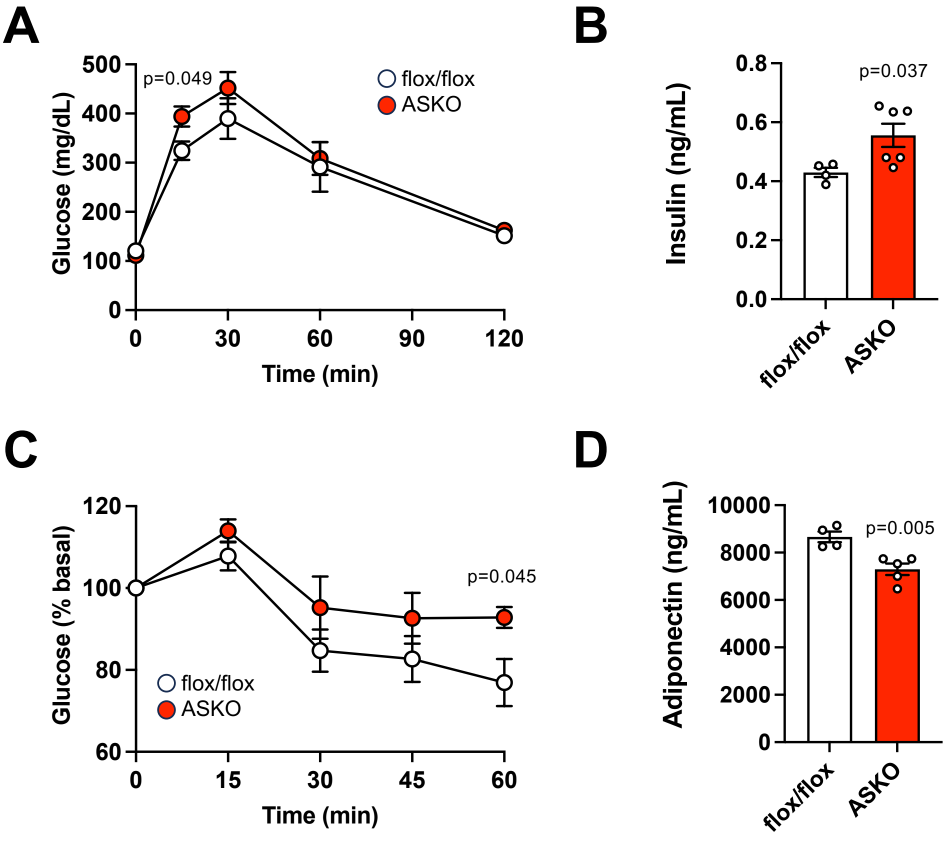

Supplemental Figure 3. Metabolic phenotype in female flox/flox and ASKO mice

Glucose metabolism was evaluated in 13 to 20-month-old female mice. Blood glucose (A) and fasted plasma insulin concentrations (B) during intraperitoneal glucose tolerance tests (IPGTTs). (C) Blood glucose concentration during insulin tolerance tests (ITTs) (n=4-7 per group). (**D**) Plasma adiponectin concentration (n=4-7 per group). Values are means ± SEM. Data were analyzed by Student's unpaired t test.

Supplemental Figure 4

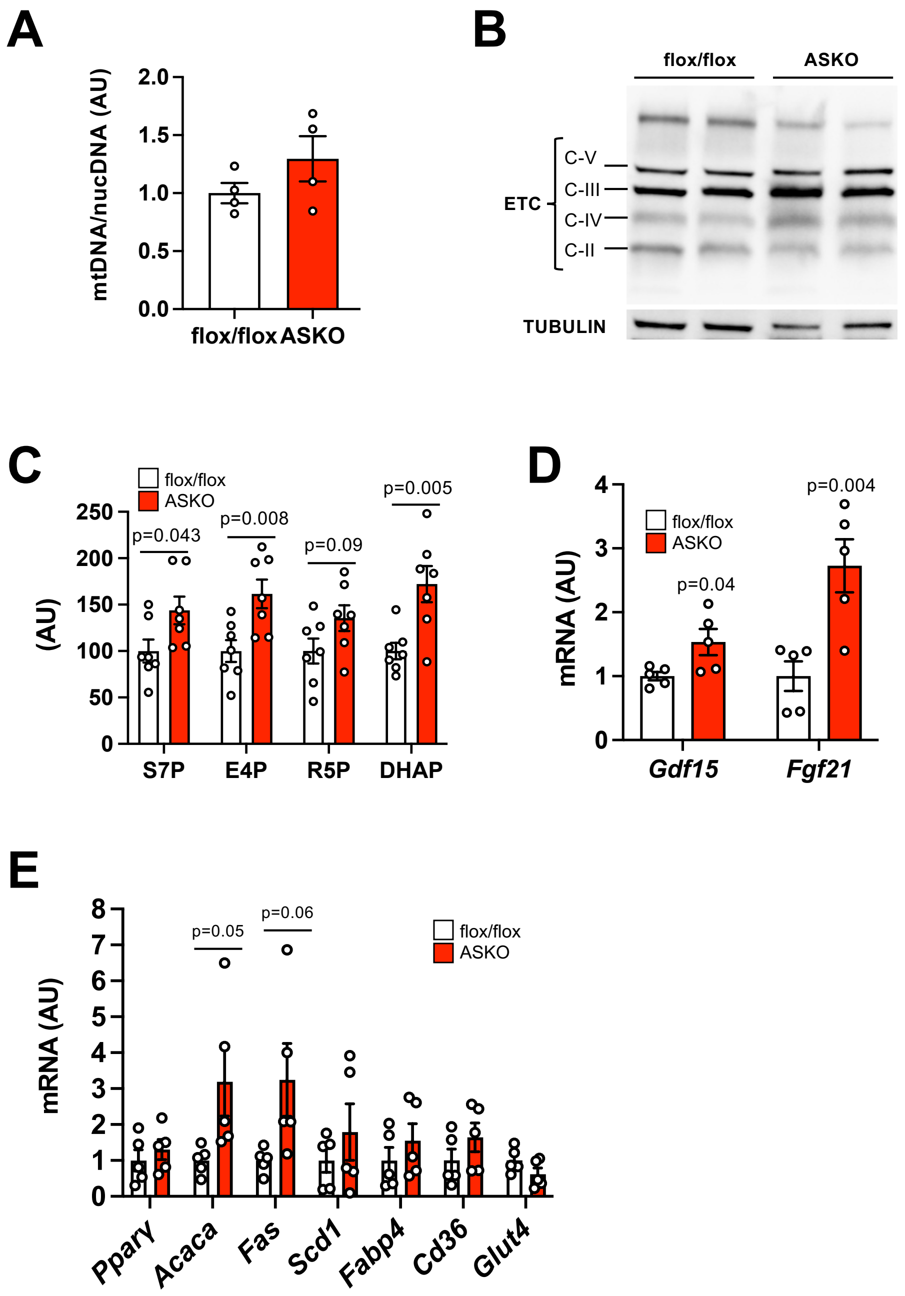

Supplemental Figure 4. Loss of SLC25A51 induced mitochondrial dysfunction and stress in adipose tissue

(**A**) Mitochondrial DNA content in iWAT (n=4 per group). (**B**) Western blot analysis of the subunits of electron transport chain (ETC) complex in iWAT. (**C**) Metabolites in pentose phosphate pathway in iWAT (n=7 per group). R5P; ribose-5-phosphate, E4P; erythrose-4-phosphate, S7P; sedoheptulose-7-phosphate, DHAP; dihydroxyacetone phosphate. (**D**) Gene expression of mitochondrial stress markers, growth

differentiation factor 15 (GDF15) and fibroblast growth factor 21 (FGF21), in iWAT (n=5 per group). (**E**) Gene expression of peroxisome proliferator-activated receptor gamma (PPARG) lipogenic targets in iWAT (n=5 per group). Values are means ± SEM. Data were analyzed by Student's unpaired t test. *Pparγ*, peroxisome proliferator-activated receptor gamma; *Acaca*, acetyl-CoA carboxylase; *Fas*, fatty acid synthase; *Scd1*, stearoyl-CoA desaturase 1; *Fabp4*; fatty acid binding protein 4; *Cd36*, cluster of differentiation 36; *Glut4*, glucose transporter 4.

Supplemental Figure 5

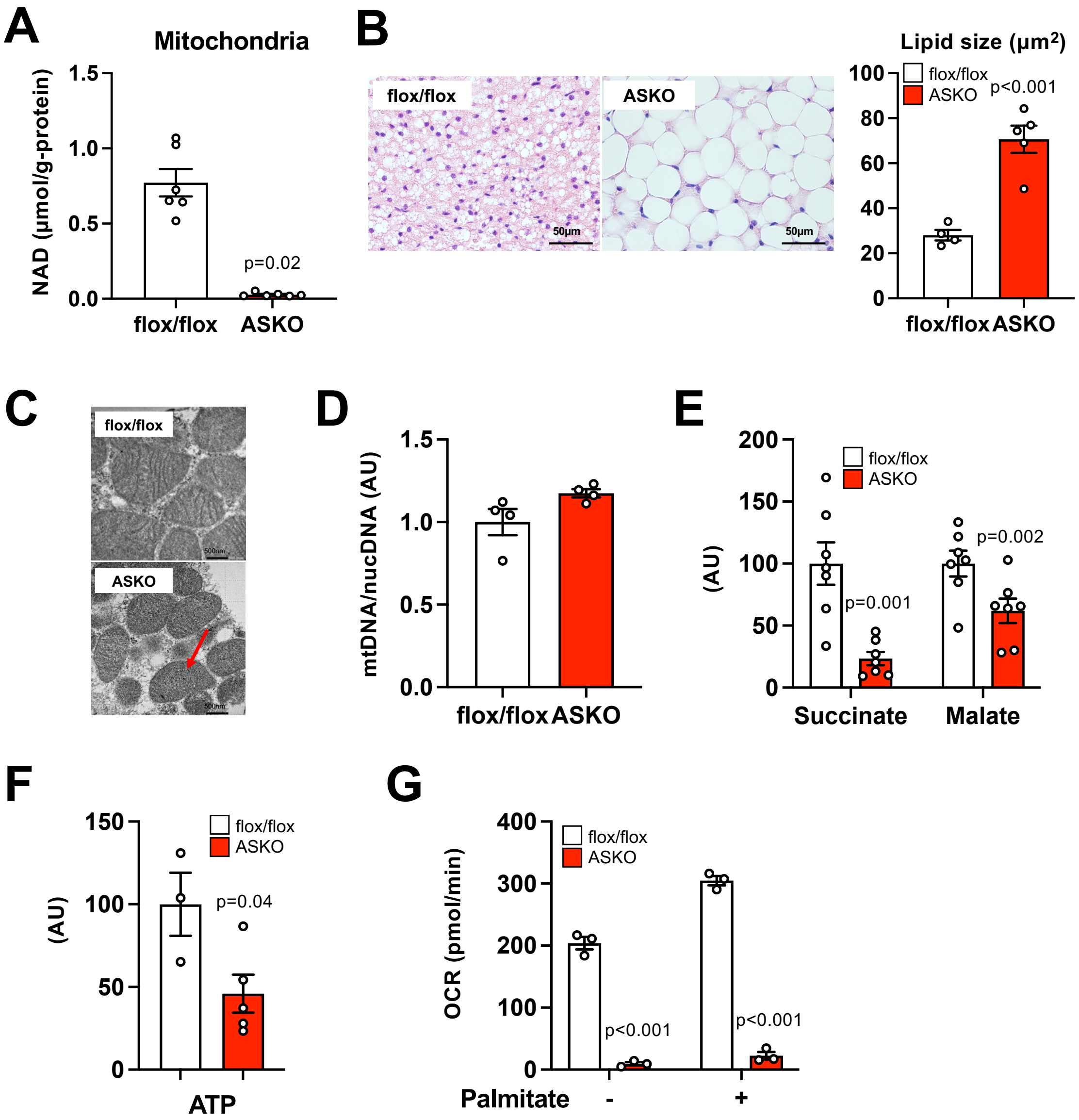

Supplemental Figure 5. The effects of Slc25a51 deletion on mitochondrial function in brown adipose tissue (BAT).

(**A**) Mitochondrial NAD concentrations in BAT (n=6 per group). (**B**) Hematoxylin and eosin-stained sections and quantification of lipid sizes (n=5 per group). (**C**) An electron microscopical study in BAT. (**D**) Mitochondrial DNA content in BAT (n=5 per group). Key metabolites in TCA cycle (**E**) and ATP (**F**) were measured in BAT (n=3-7 per group). (**G**) *Ex vivo* respiratory function was determined by the Seahorse system (n=3 per group). Oxygen consumption rates (OCR) at the baseline and responses to palmitate stimulation. Values are means ± SEM. Data were analyzed by Student's unpaired t test.

Supplemental Figure 6

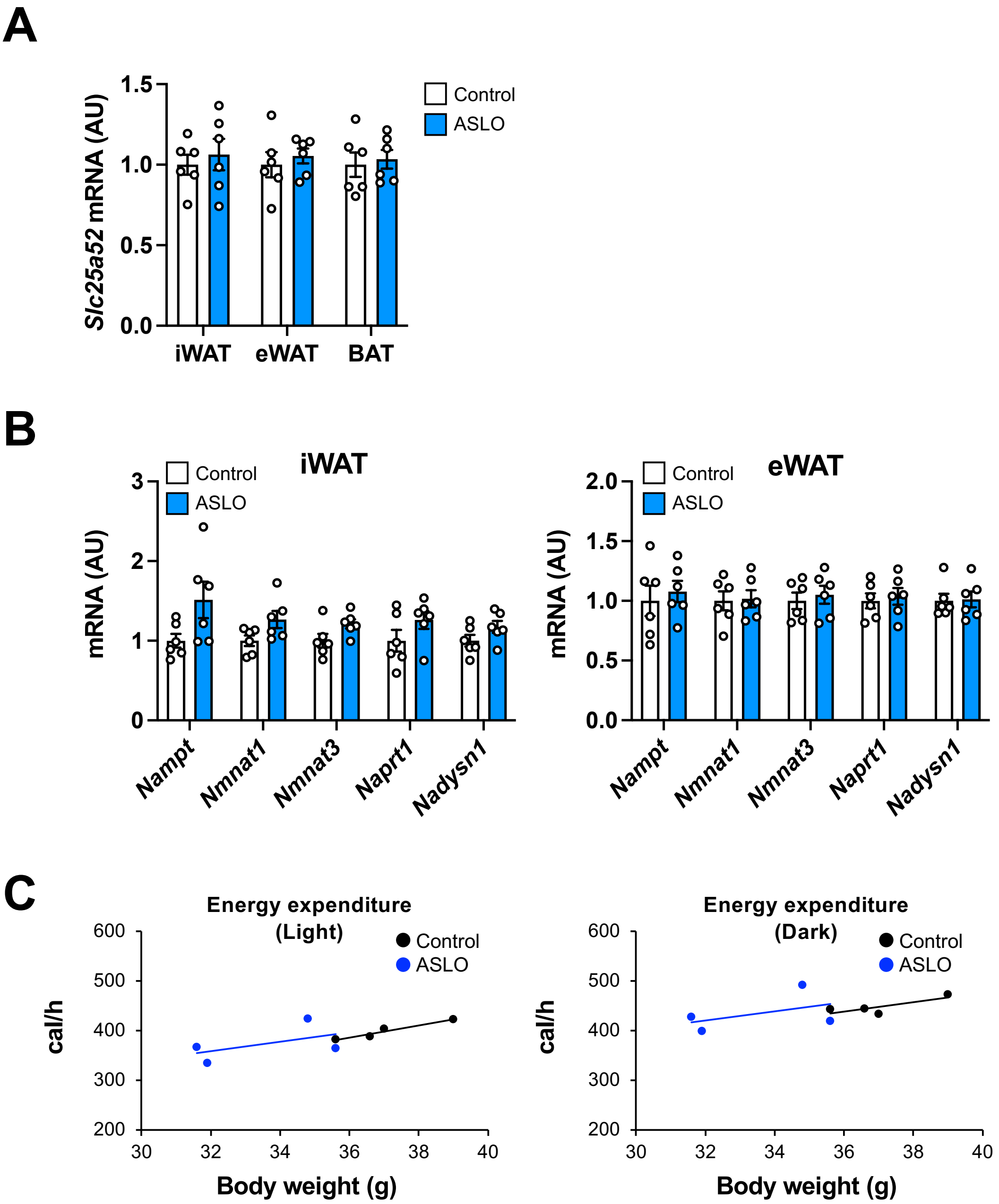

Supplemental Figure 6. Adipose tissue gene expression of enzymes involved in NAD metabolism and energy expenditure in flox/flox and adipocyte-specific *Slc25a51* overexpressing (ASLO) mice
(**A**) *Slc25a52* expression in inguinal white adipose tissue (iWAT), epididymal white adipose tissue (eWAT), and brown adipose tissue (BAT) (n=6 per group). (**B**) Gene expression of NAD biosynthetic enzymes in iWAT and eWAT (n=6 per group). (**C**) Energy expenditure (EE) was determined by the indirect calorimetry (n=4 per group). EE values were adjusted for body mass by Analysis of Covariance (ANCOVA). Values are means ± SEM. Data were analyzed by Student's unpaired t test.

Supplemental Figure 7

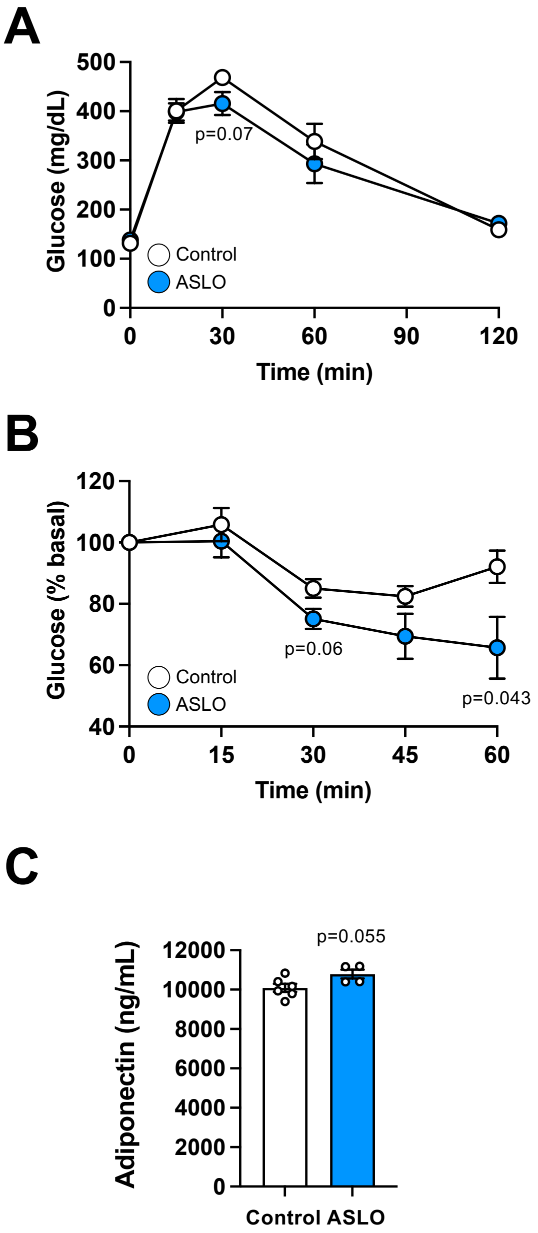

Supplemental Figure 7. Metabolic phenotype in old female flox/flox and ASLO mice

Glucose metabolism was evaluated in old (19 to 20-month-old) female mice. Blood glucose concentrations during intraperitoneal glucose tolerance tests (IPGTTs) (**A**) and insulin tolerance tests (ITTs) (**B**) (n=4-6 per group). (**C**) Plasma adiponectin concentration (n=4-6 per group). Values are means ± SEM. Data were analyzed by Student's unpaired t test.
